# MPRAVarDB: an online database and web server for exploring regulatory effects of genetic variants

**DOI:** 10.1093/bioinformatics/btae578

**Published:** 2024-09-26

**Authors:** Weijia Jin, Yi Xia, Javlon Nizomov, Yunlong Liu, Zhigang Li, Qing Lu, Li Chen

**Affiliations:** Department of Biostatistics, University of Florida, Gainesville, FL 32603, United States; Department of Biostatistics, University of Florida, Gainesville, FL 32603, United States; Department of Biostatistics, University of Florida, Gainesville, FL 32603, United States; Department of Medical and Molecular Genetics, Indiana University School of Medicine, Indianapolis, IN 46202, United States; Department of Biostatistics, University of Florida, Gainesville, FL 32603, United States; Department of Biostatistics, University of Florida, Gainesville, FL 32603, United States; Department of Biostatistics, University of Florida, Gainesville, FL 32603, United States

## Abstract

**Summary:**

Massively parallel reporter assay (MPRA) is an important technology for evaluating the impact of genetic variants on gene regulation. Here, we present MPRAVarDB, an online database and web server for exploring regulatory effects of genetic variants. MPRAVarDB harbors 18 MPRA experiments designed to assess the regulatory effects of genetic variants associated with GWAS loci, eQTLs, and genomic features, totaling 242 818 variants tested more than 30 cell lines and 30 human diseases or traits. MPRAVarDB enables users to query MPRA variants by genomic region, disease and cell line, or any combination of these parameters. Notably, MPRAVarDB offers a suite of pretrained machine-learning models tailored to the specific disease and cell line, facilitating the prediction of regulatory variants. The user-friendly interface allows users to receive query and prediction results with just a few clicks.

**Availability and implementation:**

https://mpravardb.rc.ufl.edu

## 1 Introduction

Genome-wide association studies (GWAS) and expression quantitative trait loci (eQTL) analysis have successfully identified thousands of loci associated with human diseases and traits, most of which are located within the noncoding region. However, the lead variants within these loci are not necessarily the causal variants. Instead, variants in tight linkage disequilibrium (LD) with the lead variants may be the causal ones. To address this challenge, massively parallel reporter assays (MPRAs) have been developed, which can perform high-throughput functional screen on tens of thousands of genetic variants in tight LD with selected GWAS SNPs (single nucleotide polymorphisms) or eQTLs to evaluate their impact on gene expression. Since their development, MPRAs have been widely used to test the regulatory effects of genetic variants in tight LD with GWAS SNPs, which are associated with neurodegenerative disease (Cooper *et al.* 2022), immune diseases (Mouri *et al.* 2022), various cancer types such as melanoma and multiple myeloma (Ajore *et al.* 2022, Long *et al.* 2022). Additionally, MPRAs have been performed to evaluate the regulatory effects of genetic variants in tight LD with eQTLs identified in GM12878 from the CEU cohort (Tewhey *et al.* 2016, Abell *et al.* 2022) and rare variants from multiple tissues in GTEx (Ferraro *et al.* 2020). MPRAs are particularly useful for experimentally testing the allelic difference in regulatory effects between alleles (e.g. protective and risk) of targeted variants in a single experiment. Variants showing significant allelic regulatory activity can be deemed causal variants (Tewhey *et al.* 2016). Identifying these causal variants, along with their associated risk genes, is a fundamental initial step toward developing a mechanistic understanding that may lead to therapeutic development.

Due to the rapid development of MPRAs, the volume of MPRA data has grown significantly. However, there is a lack of a centralized repository that can curate, integrate, and provide an interactive platform for querying, retrieving, downloading, and performing in-depth analysis on this data. Existing data repositories such as NCBI Gene Expression Omnibus (GEO), only offer functionality for direct data deposition and do not provide tools for interactively exploring the MPRA data. At the time of writing this manuscript, a web interface named MPRAbase (Zhao *et al.* 2023) provides only the GEO accession number and PubMed ID of MPRA studies, along with saturation mutagenesis from promoter and enhancers of approximately 20 genes from one MPRA study (Kircher *et al.* 2019). However, MPRAbase lacks detailed curation of variant annotation such as variant information and statistical measurement for the variants. It is also does not offer facilities to browse, retrieve, and download these MPRA summary data, let along perform functional screen for novel findings. To fill this gap, we introduce MPRAVarDB, an online database and web server, designed to meet the pressing need for integrating and exploring experimentally validated genetic variants from MPRA studies in public domain. MPRAVarDB houses 18 MPRA experiments that evaluate the regulatory effects of variants focusing on SNPs in GWAS loci, eQTLs, and genomic features (e.g. 5′UTR, 3′UTR), encompassing a total of 242 818 variants tested across more than 30 cell lines and 30 human diseases or traits. MPRAVarDB enables users to query MPRA variants by genomic region, disease and cell line of interest, or by any combination of these terms. Importantly, MPRAVarDB offers a set of pretrained machine-learning models tailored to specific diseases and cell line, facilitating the prediction of regulatory variants. MPRAVarDB is user-friendly, allowing users to obtain query and prediction results with just a few clicks. To the best of our knowledge, MPRAVarDB is the first and most comprehensive web tool developed for this purpose, providing significant benefits to the genetics research community.

## 2 Methods

**Figure 1.**
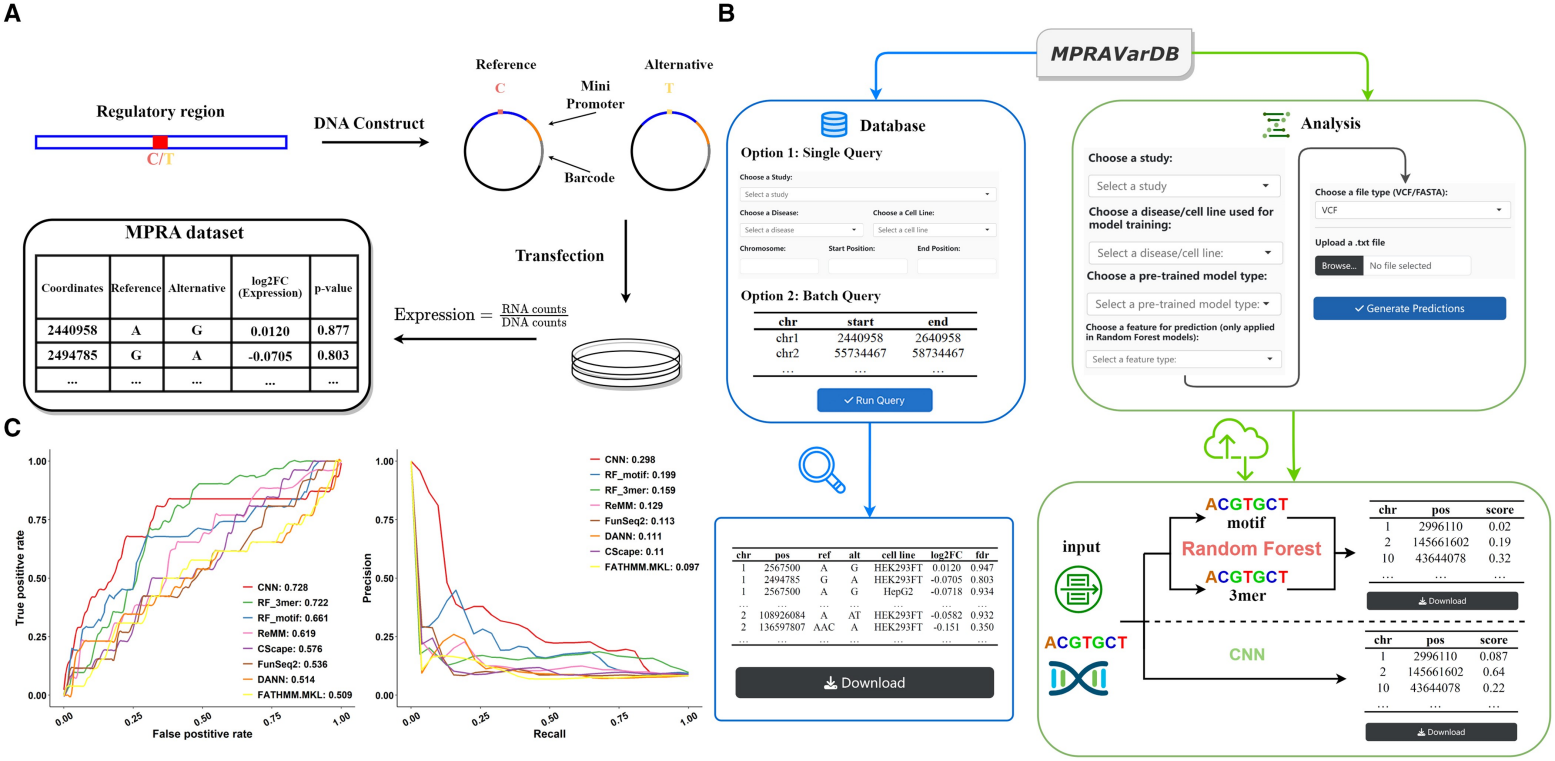
Data preparation, web server, and example for illustrating MPRAVarDB. (A) A brief introduction of MPRA experiment and summary data. MPRA experiments begin with constructing synthetic DNA constructs that include regulatory sequences harboring a short polymorphism (e.g. SNP), a minimal promoter, and a unique DNA barcode that can be transcribed. Both reference and alternative alleles of the variant are incorporated into the DNA constructs, which are further transfected into cultured cells. The surrounding regulatory sequence of the variant may possess the ability to regulate the transcription of the barcode sequence, and each allele may have different ability for the regulation. After performing RNA and DNA sequencing in the cultured cells, the transcription level for each allele is typically estimated as the ratio of RNA counts to DNA counts. Statistical analysis is conducted to compare the transcriptional activity between the two alleles, which results in a fold change between the transcriptional activity and adjusted *P*-value, indicating whether the difference is statistically significant. Variants with significant allelic regulatory effects are deemed “causal variants.” (B) Overview of MPRAVarDB. MPRAVarDB consists of two core modules: the database module and the analysis module. The database module aggregates processed MPRA summary data from publicly available sources and provide the graphical user interface for users to query and obtain the MPRA variants of interest. The analysis module focuses on providing timely prediction for the regulatory effects of query variants using pretrained machine learning models, which include two versions of random forest and convolutional neural network. These models are developed based on the labeled MPRA variants curated in the database module. (C) An example from one MPRA study focused on autoimmune diseases demonstrates the predictive power of the pretrained machine learning models compared to competing methods is provided, highlighting the effectiveness of MPRAVarDB in predicting regulatory effects of genetic variants.

MPRAVarDB consists of two core modules: the database module and the analysis module. The database module aggregates the processed MPRA summary data from publicly available MPRA experiments, allowing users to query and download the MPRA variants of interest. The analysis module offers a set of pretrained machine-learning models tailored to specific diseases and cell line, which are developed using the variants curated in the database module. These models can predict the regulatory effects for query variants that are not yet in the database collection. The overview of MPRAVarDB is shown in Fig. 1.

### 2.1 MPRA summary data

At the time of writing, MPRAVarDB contains a total of a total of 242 818 variants across 18 MPRA experiments from more than 30 cell lines and 30 human diseases or traits (Supplementary Table S1). The MPRA experiments primarily target SNPs in tight LD with the GWAS SNPs associated with different diseases or traits, as well as SNPs in tight LD with eQTLs. Each MPRA summary data has been retrieved from the original publications and processed within a unified framework. Details of data collection and processing can be found in the Supplementary Notes. Consequently, two categorical annotations are provided for each variant (Supplementary Table S2).

- Variant annotation: genomic coordinate; rsID; reference and alternative allele; genome assembly; description of MPRA variants; description of MPRA study.
- Statistical measurement: log2 fold change, *P*-value, and false discovery rate (FDR) of allelic regulatory effect.

### 2.2 Pretrained machine learning models

We provide three pretrained machine learning models, which include convolutional neural network (CNN) and two versions of random forest of different feature sets, denoted as RF_3mer and RF_motif, to predict the MPRA signals for any given variants. These models are essentially binary classifiers, which enable the identification of novel variants with high MPRA signals, even they are not represented in the training set. For the prediction models, the training set is derived from the processed MPRA summary data based on the selection of MPRA study, cell line, and disease. For MPRA studies involving multiple diseases or cell lines, we stratify the training data by disease or cell line and train independent models. The positive set includes causal variants deemed as MPRA variants with an FDR below 0.1 and absolute value of log2 fold change larger than 0.1, whereas the negative set consists of MPRA variants with an FDR exceeding 0.8. Therefore, the labels of MPRA variants serve as the binary outcome for the prediction models, and the predictions are the probability of being regulatory. The default threshold is 0.5 to determine the regulatory variants, while the users can also specify the threshold based on the prior knowledge. Moreover, the input features consist of 1000 bp flanking DNA sequences of MPRA variants with the variants in the middle of the sequences, which is further one-hot encoded. CNN then employs the one-hot encoding DNA sequences as the input features. RF_3mer and RF_motif differ in their strategies for extracting features from the flanking DNA sequences. RF_3mer uses the 3-mer frequency of the DNA sequence, which results in 64 features. RF_motif utilizes the frequency of 633 human motifs (Supplementary Notes). We adopt a chromosome-split strategy to create training, validation, and testing sets. We sequentially include MPRA variants on chromosome order, ensuring the proportions of MPRA variants in the training, validation, and testing sets are approximately 60%:20%:20%. Additionally, both positive and negative strands of DNA sequences are used in the model training to augment the sample size.

### 2.3 Implementation

MPRAVarDB is written in R Shiny. The R Shiny interface is developed and hosted on a PubApp instance from HiPerGator supercomputer at University of Florida. The PubApp instance consists of two CPU cores from AMD EPYC 75F3 Milan 3.0, with each core with 8GM RAM. CNN is implemented using PyTorch on an NVIDIA A100 GPU system. Mini-batch gradient descent is adopted for training the network and the Adam optimizer is used for minimizing cross-entropy loss with a default learning rate is $10^{-3}$. Each model undergoes training for a maximum of 200 epochs, with early stopping implemented if the model performance stagnated over 10 consecutive epochs. The hyperparameters of the CNN are fine-tuned independently for each model. Random forest is trained using “randomForest” function in R package “randomForest” with default parameter settings.

### 2.4 Workflow for database module

- Data preparation: There are two options for exploring the database. For the “Single Query” option, MPRA study, disease, and cell line can be selected from the dropdown list of “Choose a study,” “Choose a disease,” and “Choose a cell line.” The query region can be entered into three text boxes named “Chromosome,” “Start Position,” and “End Position.” For the “Batch Query” option, a bed file can be uploaded, which contains multiple rows with each row corresponding to a genomic region. Each genomic region consists of three required fields “chromosome,” “start,” and “end” and two optional columns “disease” and “cell line.”
- Data upload: For the “Batch Query” option, the bed file can be uploaded by the “Browse” and “Load File” two buttons. A preview option is provided to check the validity of the file format.
- Select disease and cell line: For the “Single Query” option, the MPRA study, disease, and cell line can be selected from the dropdown list. For the “Batch Query” option, the disease and cell line can be added as two optional columns in the bed file.
- Results display and download: For the “Single query” option, once selections are made and the “Run Query” button is clicked, the results will be displayed below in a table. For the “Batch Query” option, once the bed file has been uploaded and the “Run Query” button is clicked, the results will be displayed below in a table. For both options, once the results are displayed, a “Download” button will show up for allowing the users to download the query results.

### 2.5 Workflow for analysis module

- Model preparation: The pretrained model is selected based on the choice of reference genome, MPRA study, disease, cell line, and types of machine learning models, which can be selected from the dropdown list of “Choose a genome,” “Choose a study,” and “Choose a category for stratifying the training data,” “Choose a disease/cell line for model training,” “Choose a model type,” “Choose a feature for prediction (only applied to Random Forest),” and “Choose a file type (VCF/FASTA).”
- Data preparation: Two formats of query data are supported: (i) a vcf file, which contains multiple variants. Each row corresponds to the genomic coordinate of one query variant, which consists of two required fields “chromosome” and “position” and (ii) a fasta file, which includes multiple DNA sequences. Each DNA sequence can be selected surrounding one query variant with a length of 1000 bp. Clicking the “Browse” button will upload the query data from the local computer to the online server.
- Data upload: The vcf or fasta file can be uploaded by the “Browse” and “Load File” two buttons. A preview option is provided to check the validity of the file format.
- Prediction, results display, and download: Clicking “Generation Prediction” will call the selected pretrained machine learning model to predict the regulatory effects of query variants, which will generate a table with prediction scores. A “Download” button is also provided to download the tables.

## 3 Examples

To demonstrate the practical application of the analysis module, we evaluate CNN, RF_3mer, and RF_motif in several MPRA experiments. As a benchmark, we collect the genome-wide precomputed functional score from five variant scoring methods: CScape (Rogers *et al.* 2017), FATHMM_MKL (Shihab *et al.* 2015), DANN (Quang *et al.* 2015), ReMM (Smedley *et al.* 2016) and FunSeq2 (Fu *et al.* 2014). We assess the prediction performance using labeled MPRA variants from an MPRA study that evaluates variants in tight LD ($r^{2}$ > 0.8) with 578 GWAS index variants representing 531 distinct GWAS loci associated with five autoimmune diseases. The training set for this evaluation includes 195 positive variants. To avoid severe class imbalance, we randomly sample ten times the number of positive variants from the pool of 12 664 negative variants to include as the negative variants in the training set. Consequently, CNN performs best by achieving the highest AUROC (Area Under the Receiver Operating Characteristic Curve) and AUPRC (Area Under the Precision-Recall Curve; Fig. 1C). Additionally, we evaluate on labeled MPRA variants targeting variants in LD within 25 genome-wide significant loci associated with Alzheimer’s disease (AD) and nine loci associated with Progressive Supranuclear Palsy (PSP; Cooper *et al.* 2022), comprising 201 positive and 655 negative variants for AD, and 410 positive and 1352 negative variants for PSP, respectively. Another example includes 242 positive and 1109 negative variants in tight LD ($r^{2}$ > 0.8) from 54 risk loci associated with melanoma (Long *et al.* 2022). The final example involves labeled MPRA variants profiled in HMEC cell line from a MPRA study, which performs a genome-wide functional screens of 3′UTR variants for human disease and evolution (Griesemer *et al.* 2021). The MPRA study investigates three sets of variants: disease-associated 3′UTR SNPs in LD from the NHGRI-EBI GWAS catalog, 3′UTR SNPs in regions under positive selection, and rare 3′UTR SNPs in genes with outlier expression signatures across tissues in GTEx, which consists of 1188 positive and 4207 negative variants. All results for the additional four comparisons are demonstrated in Supplementary Figs. S1–S4, where we observe that CNN and RF_3mer lead the performance in most cases (Supplementary Tables S3 and S4). These observations demonstrate that the advantage of using pretrained machine learning methods in MPRAVarDB’s analysis module in predicting MPRA signals by leveraging disease- and cell line-specific labeled MPRA variants in the MPRAVarDB’s database module.

## 4 Discussion

In this work, we present MPRAVarDB, an online database and web server that facilitates the exploration of MPRA variants associated with different diseases and cell lines from a wide collection of MPRA studies. The database module of MPRAVarDB features a user-friendly interface that allows users to query arbitrary genomic regions. Notably, the analysis module provides a comprehensive set of pretrained machine learning models, developed using labeled MPRA variants from the database module, to predict regulatory efforts for any given variants. This functionality empowers the discovery of novel regulatory variants that are not yet in the training set. Therefore, MPRAVarDB significantly reduces the obstacles for scientists to retrieve and utilize MPRA variants from public domains. We plan to incorporate more public data as they become available and update the pretrained machine learning models accordingly. Our ultimate goal is to turn MPRAVarDB into a powerful toolbox that can efficiently integrate publicly available MPRA variants into a unified framework and make novel prediction on the regulatory effects of variants beyond the experimentally validated ones.

## Supplementary Material

btae578_Supplementary_Data
