# Supplementary material for: MPRAVarDB: an online database and web server for exploring regulatory effects of genetic variants
Source: Bioinformatics. 2024 Sep 26;40(10):btae578. doi: 10.1093/bioinformatics/btae578 (PMC11464417; doi:10.1093/bioinformatics/btae578)
Supplement: btae578_Supplementary_Data [file btae578_supplementary_data.pdf]

Supplementary Materials for

MPRAVarDB: a database and web server for exploring regulatory effects of genetic variants

Supplementary table

Table S1: Summary of 18 MPRA studies

Study	disease	Cell line	Description	System	treatment	Replicates
Functional regulatory variants implicate distinct transcriptional networks in dementia (Cooper et al., 2022)	Alzheimer's disease (AD) and Progressive supranuclear palsy (PSP)	HEK293T	5706 noncoding single-nucleotide variants, representing 25 genome-wide significant loci associated with AD and nine loci associated with PSP	transient	none	6 technical replicates
Multiple causal variants underlie genetic associations in humans (Abell et al., 2022)	None	GM12878	30,893 variants in LD with selected independent, common, and top-ranked eQTL across 744 eGenes identified in the CEU cohort	transient	none	3 biological replicates
Transcriptomic signatures across human tissues identify functional rare genetic variation (Ferraro et al., 2020)	None	GM12878	Rare genetic variants contributing to extreme patterns in gene expression, allelic expression, and alternative splicing across 49 tissues in GTEx	transient	none	3 biological replicates
Genome-wide functional screen of 3'UTR variants uncovers causal variants for human disease and evolution (Griesemer et al., 2021)	NHGRI-EBI GWAS catalog	HMEC,HEK293FT,HEPG2,K562,GM12878,SKNSH	3' UTR SNPs and insertion/ deletions (indels) in LD from the NHGRI-EBI GWAS catalog, which include 2,153 putative disease-associated variants including from 1,556 independent association loci; 9,325 3'UTR SNPs and indels overlapping regions identified	transient	none	4-5 replicates

			as being under positive selection in humans; a set of 46 rare 3’UTR variants in genes with outlier expression signatures across tissues in GTEx.			
Functional testing of thousands of osteoarthritis-associated variants for regulatory activity (Klein et al., 2019)	Osteoarthritis	Saos-2	1,605 SNPs in high LD (r2 > 0.8) at 35 lead SNPs associated with OA in Europeans via GWAS	transient	none	3-4 biological replicates
Transcriptional-regulatory convergence across functional MDD risk variants identified by massively parallel reporter assays (Mulvey & Dougherty, 2021)	Major depressive disorder (MDD)	N2A	Over 1000 SNPs are selected from 39 neuropsychiatric trait/disease GWAS loci, which is based on overlap with eQTL and histone marks from human brains and cell cultures.	transient	none	6 replicates
Saturation mutagenesis of twenty disease-associated regulatory elements at single base-pair resolution (Kircher et al., 2019)	~20 diseases and details can be found at https://kircherlab.bihealth.org/satMutMPRA/	HepG2 (HB-8065), HEK293T (CRL-11268), HeLa (CCL-2), HaCaT (CRL-2404), Neuro-2a (CCL-131), LNCaP (CRL-1740), and SK-MEL-28 (HTB-72)	Over 30,000 single nucleotide substitutions and deletions in 20 disease-associated gene promoters and enhancers	transient	none	2 biological replicates
Functional dissection of inherited non-coding variation influencing multiple myeloma risk (Ajore et al., 2022)	Multiple myeloma (MM)	L363, MOLP8	1039 variants in high LD (r2 > 0.8) at 23 MM risk loci	transient	none	3 biological replicates
Global discovery of lupus genetic risk variant allelic enhancer activity (Lu et al., 2021)	Systemic Lupus Erythematosus (SLE)	GM12878, Jurkat T cell	3073 variants in high LD (r2 > 0.8) at 91 SLE-associated risk loci	transient	none	3 biological replicates

Prioritization of autoimmune disease-associated genetic variants that perturb regulatory element activity in T cells UTR	Five Autoimmune diseases: Type 1 diabetes (T1D), Ulcerative colitis (UC),Crohn's disease (CD), Rheumatoid arthritis (RA), Multiple sclerosis (MS).	Jurkat T cell	18,312 variants in tight LD (r2>0.8) with 578 GWAS index variants representing 531 distinct GWAS loci.	transient	none	2 biological replicates
Massively parallel reporter assays and variant scoring identified functional variants and target genes for melanoma loci and highlighted cell-type specificity (Long et al., 2022)	Melanoma	UACC903 (malignant melanoma) C283T (normal melanocyte cells)	1,992 risk-associated variants in tight LD (r2>0.8) from 54 risk-associated loci	transient	none	>=3 biological replicates
Massively parallel reporter assays of melanoma risk variants identify MX2 as a gene promoting melanoma (Choi et al., 2020)	Melanoma	UACC903, HEK293FT	832 high-LD variants at melanoma susceptibility GWAS loci	transient	none	3 biological replicates
A screen of 1049 schizophrenia and 30 Alzheimer's-associated variants for regulatory potential (Myint et al., 2020)	Alzheimer's disease and Schizophrenia	K562,SK-SY5Y	1,049 SZ and 30 AD variants in 64 SZ loci and 9 AD loci, respectively	transient	none	5 technical replicates
Systematic Functional Dissection of Common Genetic Variation Affecting Red Blood Cell Traits (Ulirsch et al., 2016)	Red Blood Cell Traits	K562, K562+GATA1	2,756 variants in strong linkage disequilibrium with 75 sentinel variants associated with red blood cell traits	transient	none	6 replicates for K562 cells and 4 replicates for K562+GATA1 cells
Direct Identification of Hundreds of Expression-Modulating Variants using a Multiplexed Reporter Assay (Tewhey et al., 2016)	NA	GM12878	32,373 variants associated with eQTLs in lymphoblastoid cell lines	transient	none	8 technical replicates for the lymphoblastoid and 5 for hepato-carcinoma cell lines
Allele-specific expression and high-throughput reporter assay reveal functional genetic variants associated with alcohol use disorders (Rao et al., 2021)	Alcohol use disorders	SH-SY5Y and SK-N-BE(2)	SNPs in 3'UTR of 88 genes identified from allele-specific expression analysis between 30 subjects with Alcohol use disorders and 30 controls.	transient	ethanol	4 biological replicates
Multi-level functional genomics reveals molecular and cellular oncogenicity of patient-based 3' untranslated	Prostate cancer	PC3 cell	14,497 single-nucleotide mutations enriched in oncogenic	transient	None	6 biological replicates

region mutations(Schuster et al., 2023)			pathways and 3' UTR regulatory elements.			
Systematic investigation of allelic regulatory activity of schizophrenia-associated common variants (McAfee et al., 2023)	Schizophrenia	HEK293s, HNPS	5,173 fine-mapped schizophrenia GWAS variants	transient	none	10 biological replicates

Table S2: Summary of 13 variant annotations

Column name	Interpretation
chr	chromosome of the variant
pos	genomic position of the variant
ref	reference allele of the variant
alt	alternative allele of the variant
genome	reference genome of the variant
rsid	SNP ID of the variant
disease	disease type from the GWAS
cell line	cell line used from MPRA experiment
description	brief description of the source of MPRA variants
log2FC	Log2 fold change of transcription level between reference and alternative allele
pvalue	pvalue of transcription level change between reference and alternative allele
fdr	fdr of transcription level change between reference and alternative allele
MPRA study	name of the MPRA study

Table S3: Comparing machine learning methods for predicting MPRA regulatory variants in terms of AUC

Disease	AUC							
	CNN	RF_motif	RF_3mer	CScape	DANN	FATHMM.MKL	FunSeq2	ReMM
AD	**0.64**	0.501	0.625	0.51	0.528	0.502	0.518	0.54
PSP	0.57	0.617	**0.659**	0.559	0.551	0.597	0.556	0.557
3'UTR(HMEC)	**0.599**	0.551	0.531	0.553	0.521	0.52	0.517	0.526
Autoimmune	**0.728**	0.661	0.722	0.576	0.514	0.509	0.536	0.619
Melanoma	**0.677**	0.567	0.644	0.545	0.581	0.496	0.544	0.517

Table S4: Comparing machine learning methods for predicting MPRA regulatory variants in terms of AUPRC

Disease/method	AUPRC							
	CNN	RF_motif	RF_3mer	CScape	DANN	FATHMM.MKL	FunSeq2	ReMM
AD	**0.495**	0.373	0.428	0.348	0.341	0.354	0.425	0.399
PSP	0.218	0.274	**0.369**	0.167	0.197	0.159	0.163	0.176
3'UTR(HMEC)	**0.286**	0.219	0.217	0.218	0.203	0.222	0.189	0.209
Autoimmune	**0.298**	0.199	0.159	0.11	0.111	0.097	0.113	0.129
Melanoma	**0.397**	0.305	0.324	0.261	0.193	0.248	0.285	0.24

Supplementary Notes

Computational pipeline to process MPRA summary data

We collect the MPRA summary data from 18 MPRA studies in **Table S1**. Next, we use a computational pipeline to process MRPA variants from all studies into the same format with 13 annotations (**Table S2**). For annotations missing from the MPRA summary data, we will adopt the following bioinformatics tools to generate the annotations. Specifically,

1. **Variant annotation:**
   If genomic position, reference and alternative alleles are provided for variants in the MPRA summary data but rsID is missing, we adopt "snpsByOverlaps" function in in "SNPlocs.Hsapiens.dbSNP144.GRCh37/GRCh38" R/Bioconductor package, which takes the "GRanges" object of query variants and reference SNPs in "SNPlocs.Hsapiens.dbSNP144.GRCh37/GRCh38" R/Bioconductor package as the parameters and output the rsID. If rsID is provided for variants in the MPRA summary data but reference and alternative alleles are missing, we adopt "snpsById" function, which takes rsID and reference SNPs in "SNPlocs.Hsapiens.dbSNP144.GRCh37/GRCh38" R/Bioconductor package as the parameters and output the genomic positions of query variants. Next, we adopt "inferRefAndAltAlleles" function in "BSgenome.Hsapiens.UCSC.hg19/hg38" R/Bioconductor package, which takes the genomic positions of query variants and human refence genome in "BSgenome.Hsapiens.UCSC.hg19/hg38" R/Bioconductor package as the parameters and output the reference and alternative alleles for the query variants.

2. **Statistical measurement:**
   If log2FC and pvalue are missing, we fill the missing entries with NA.

Feature extraction for machine learning models

1. **Motif frequency for DNA sequence**
   For random forest model with motif frequency as the feature, we obtain the motif feature in the R/Bioconductor environment. The human motifs are collected from Bioconductor package "JASPAR2020" with species ID 9606, which results in 633 Position frequency matrix (PFM). Motif scan is carried out by using the 633 PFMs to scan 1kb DNA sequence surrounding each MPRA variant with the variant in the middle. The DNA sequence can be provided in the fasta file directly or as genomic regions in the bed file. If input file is fasta file, the DNA sequences will be converted into "DNAStringSet" object in R/Bioconductor package "Biostrings". If input file is bed file, the genomic regions will be converted to "GRanges" object in R/Bioconductor package "GenomicRanges". Next, "matchMotifs" function in R/Bioconductor package "motifmatchr", if input file is fasta file, takes two parameters including (1) 633 human motifs in the format of PFMs (2) DNA sequences stored in the "DNAStringSet" object; if input file is bed file, takes three parameters including (1) 633 human motifs in the format of PFMs (2) genomic regions stored in a "GRanges" object and (3) Human reference genome represented R/Bioconductor package"BSgenome.Hsapiens.UCSC.hg19/hg38". As a result, for each DNA sequence, the motif features will be generated as a vector of 633 motif frequency.

2. **3mer frequency for DNA sequence**
   For random forest with 3mer frequency as the feature, we obtain 3mer frequency in the R/Bioconductor environment. If input file is fasta file, the DNA sequences will be converted into "DNAStringSet" object in R/Bioconductor package "Biostrings". If the input file is vcf file, the genomic regions will be converted to "GRanges" object in R/Bioconductor package "GenomicRanges". "getSeq" function in R/Bioconductor package "BSgenome", takes both the "GRanges" object and human reference genom"BSgenome.Hsapiens.UCSC.hg19/hg38" as the parameters and output the DNA sequences in a "DNAStringSet" object. Next, "trinucleotideFrequency" function in R/Bioconductor package "Biostrings" takes "DNAStringSet" object and extracts the 3mer frequency, resulting in 64 3mer features.

3. **One-hot encoding for DNA sequence**
   For the convolutional neural network (CNN), 1000bp flanking DNA sequence surrounding each MPRA variant with the variant in the middle, is one-hot encoded (A: [1,0,0,0], C: [0,1,0,0], G: [0,0,1,0] and T: [0,0,0,1]) as the feature representation for each MPRA variant.

Supplementary Figures

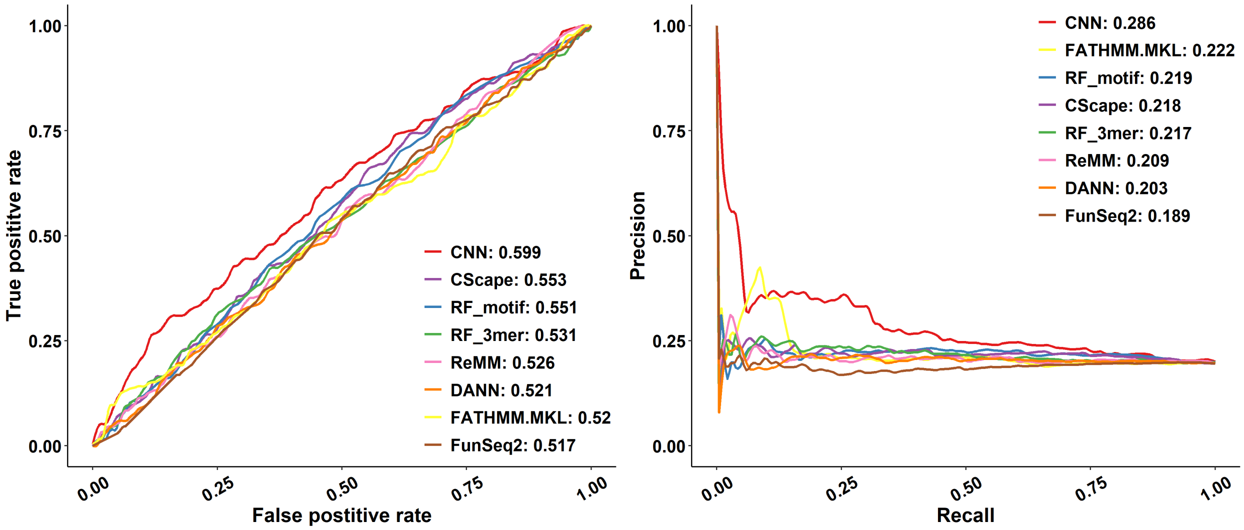

Figure S1: Comparing machine learning methods for predicting MPRA regulatory variants associated with human 3'UTR profiled by HMEC cell line in terms of AUC and AUPRC

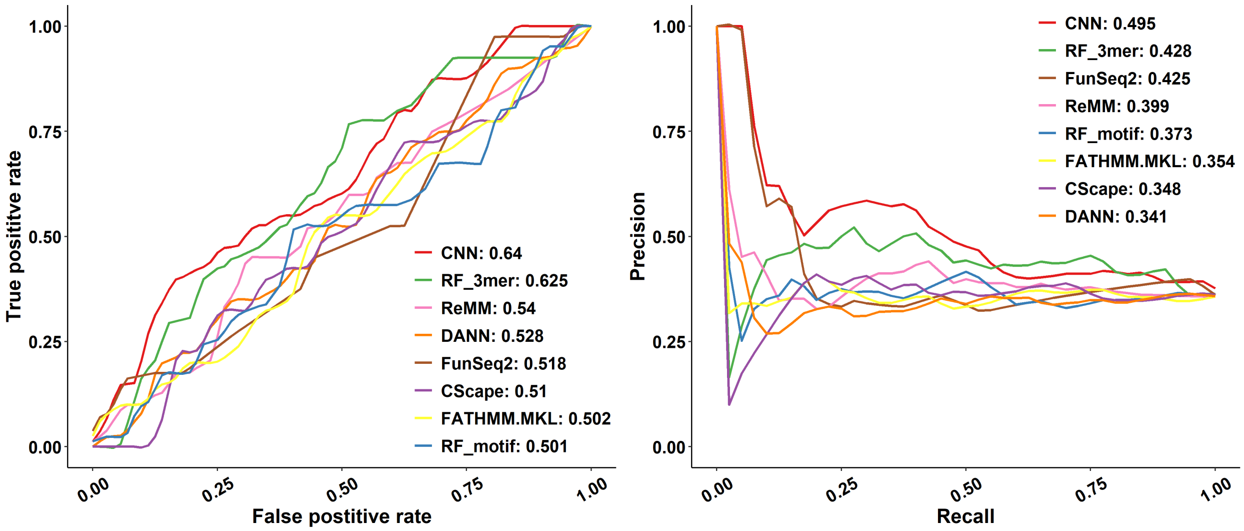

Figure S2: Comparing machine learning methods for predicting MPRA regulatory variants associated with Alzheimer's disease in terms of AUC and AUPRC

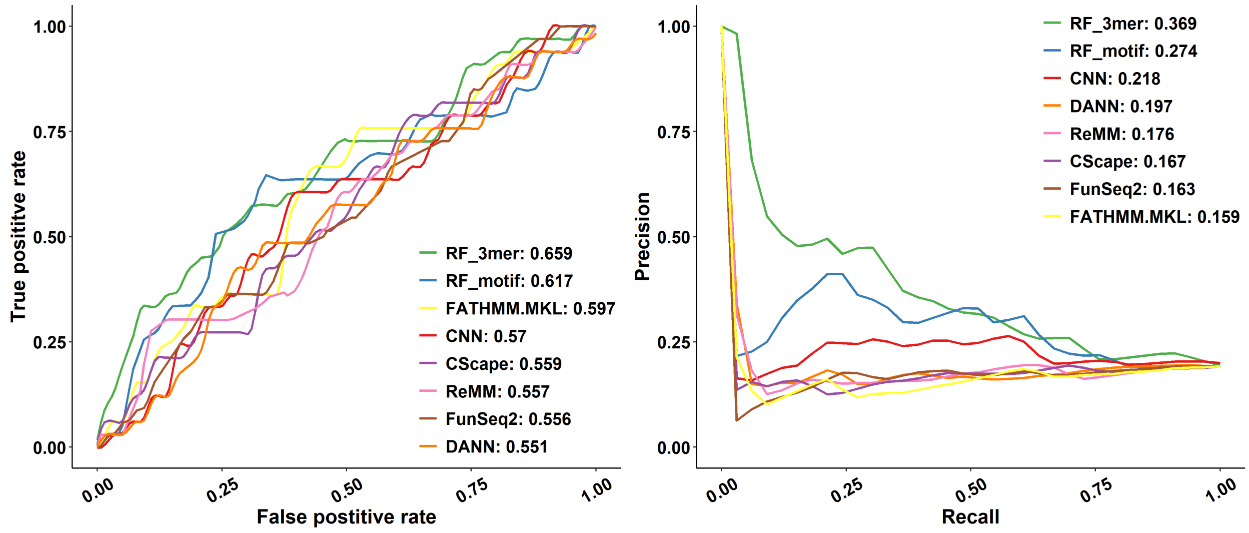

Figure S3: Comparing machine learning methods for predicting MPRA regulatory variants associated with Progressive supranuclear palsy in terms of AUC and AUPRC

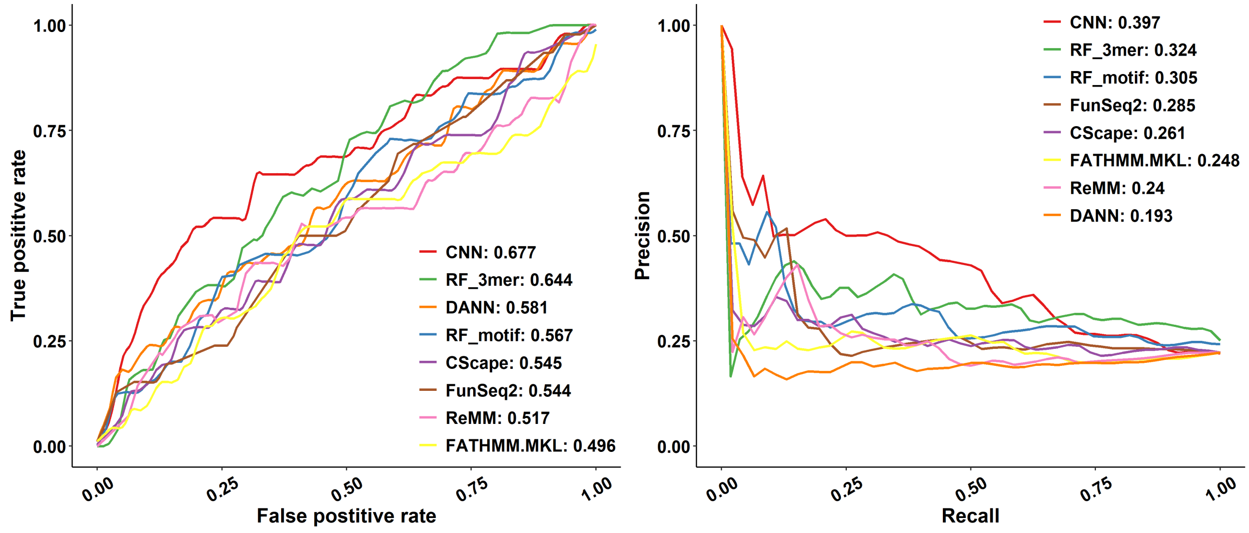

Figure S4: Comparing machine learning methods for predicting MPRA regulatory variants associated with Melanoma in terms of AUC and AUPRC

Reference

Abell, N. S., DeGorter, M. K., Gloudemans, M. J., Greenwald, E., Smith, K. S., He, Z., & Montgomery, S. B. (2022). Multiple causal variants underlie genetic associations in humans. *Science*, *375*(6586), 1247-1254. https://doi.org/10.1126/science.abj5117

Ajore, R., Niroula, A., Pertesi, M., Cafaro, C., Thodberg, M., Went, M., Bao, E. L., Duran-Lozano, L., Lopez de Lapuente Portilla, A., Olafsdottir, T., Ugidos-Damboriena, N., Magnusson, O., Samur, M., Lareau, C. A., Halldorsson, G. H., Thorleifsson, G., Norddahl, G. L., Gunnarsdottir, K., Forsti, A., . . . Nilsson, B. (2022). Functional dissection of inherited non-coding variation influencing multiple myeloma risk. *Nat Commun*, *13*(1), 151. https://doi.org/10.1038/s41467-021-27666-x

Choi, J., Zhang, T., Vu, A., Ablain, J., Makowski, M. M., Colli, L. M., Xu, M., Hennessey, R. C., Yin, J., Rothschild, H., Grawe, C., Kovacs, M. A., Funderburk, K. M., Brossard, M., Taylor, J., Pasaniuc, B., Chari, R., Chanock, S. J., Hoggart, C. J., . . . Brown, K. M. (2020). Massively parallel reporter assays of melanoma risk variants identify MX2 as a gene promoting melanoma. *Nat Commun*, *11*(1), 2718. https://doi.org/10.1038/s41467-020-16590-1

Cooper, Y. A., Teyssier, N., Drager, N. M., Guo, Q., Davis, J. E., Sattler, S. M., Yang, Z., Patel, A., Wu, S., Kosuri, S., Coppola, G., Kampmann, M., & Geschwind, D. H. (2022). Functional regulatory variants implicate distinct transcriptional networks in dementia. *Science*, *377*(6608), eabi8654. https://doi.org/10.1126/science.abi8654

Ferraro, N. M., Strober, B. J., Einson, J., Abell, N. S., Aguet, F., Barbeira, A. N., Brandt, M., Bucan, M., Castel, S. E., Davis, J. R., Greenwald, E., Hess, G. T., Hilliard, A. T., Kember, R. L., Kotis, B., Park, Y., Peloso, G., Ramdas, S., Scott, A. J., . . . Battle, A. (2020). Transcriptomic signatures across human tissues identify functional rare genetic variation. *Science*, *369*(6509). https://doi.org/10.1126/science.aaz5900

Griesemer, D., Xue, J. R., Reilly, S. K., Ulirsch, J. C., Kukreja, K., Davis, J. R., Kanai, M., Yang, D. K., Butts, J. C., Guney, M. H., Luban, J., Montgomery, S. B., Finucane, H. K., Novina, C. D., Tewhey, R., & Sabeti, P. C. (2021). Genome-wide functional screen of 3'UTR variants uncovers causal variants for human disease and evolution. *Cell*, *184*(20), 5247-5260 e5219. https://doi.org/10.1016/j.cell.2021.08.025

Kircher, M., Xiong, C., Martin, B., Schubach, M., Inoue, F., Bell, R. J. A., Costello, J. F., Shendure, J., & Ahituv, N. (2019). Saturation mutagenesis of twenty disease-associated regulatory elements at single base-pair resolution. *Nat Commun*, *10*(1), 3583. https://doi.org/10.1038/s41467-019-11526-w

Klein, J. C., Keith, A., Rice, S. J., Shepherd, C., Agarwal, V., Loughlin, J., & Shendure, J. (2019). Functional testing of thousands of osteoarthritis-associated variants for regulatory activity. *Nat Commun*, *10*(1), 2434. https://doi.org/10.1038/s41467-019-10439-y

Long, E., Yin, J., Funderburk, K. M., Xu, M., Feng, J., Kane, A., Zhang, T., Myers, T., Golden, A., Thakur, R., Kong, H., Jessop, L., Kim, E. Y., Jones, K., Chari, R., Machiela, M. J., Yu, K., Melanoma Meta-Analysis, C., Iles, M. M., . . . Choi, J. (2022). Massively parallel reporter assays and variant scoring identified functional variants and target genes for melanoma loci and highlighted cell-type specificity. *Am J Hum Genet*, *109*(12), 2210-2229. https://doi.org/10.1016/j.ajhg.2022.11.006

Lu, X., Chen, X., Forney, C., Donmez, O., Miller, D., Parameswaran, S., Hong, T., Huang, Y., Pujato, M., Cazares, T., Miraldi, E. R., Ray, J. P., de Boer, C. G., Harley, J. B., Weirauch, M. T., & Kottyan, L. C. (2021). Global discovery of lupus genetic risk variant allelic enhancer activity. *Nat Commun*, *12*(1), 1611. https://doi.org/10.1038/s41467-021-21854-5

McAfee, J. C., Lee, S., Lee, J., Bell, J. L., Krupa, O., Davis, J., Insigne, K., Bond, M. L., Zhao, N., Boyle, A. P., Phanstiel, D. H., Love, M. I., Stein, J. L., Ruzicka, W. B., Davila-Velderrain, J., Kosuri, S., & Won, H. (2023). Systematic investigation of allelic regulatory activity of schizophrenia-associated common variants. *Cell Genom*, *3*(10), 100404. https://doi.org/10.1016/j.xgen.2023.100404

Mulvey, B., & Dougherty, J. D. (2021). Transcriptional-regulatory convergence across functional MDD risk variants identified by massively parallel reporter assays. *Transl Psychiatry*, *11*(1), 403. https://doi.org/10.1038/s41398-021-01493-6

Myint, L., Wang, R., Boukas, L., Hansen, K. D., Goff, L. A., & Avramopoulos, D. (2020). A screen of 1,049 schizophrenia and 30 Alzheimer's-associated variants for regulatory potential. *Am J Med Genet B Neuropsychiatr Genet*, *183*(1), 61-73. https://doi.org/10.1002/ajmg.b.32761

Rao, X., Thapa, K. S., Chen, A. B., Lin, H., Gao, H., Reiter, J. L., Hargreaves, K. A., Ipe, J., Lai, D., Xuei, X., Wang, Y., Gu, H., Kapoor, M., Farris, S. P., Tischfield, J., Foroud, T., Goate, A. M., Skaar, T. C., Mayfield, R. D., . . . Liu, Y. (2021). Allele-specific expression and high-throughput reporter assay reveal functional genetic variants associated with alcohol use disorders. *Mol Psychiatry*, *26*(4), 1142-1151. https://doi.org/10.1038/s41380-019-0508-z

Schuster, S. L., Arora, S., Wladyka, C. L., Itagi, P., Corey, L., Young, D., Stackhouse, B. L., Kollath, L., Wu, Q. V., Corey, E., True, L. D., Ha, G., Paddison, P. J., & Hsieh, A. C. (2023). Multi-level functional genomics reveals molecular and cellular oncogenicity of patient-based 3' untranslated region mutations. *Cell Rep*, *42*(8), 112840. https://doi.org/10.1016/j.celrep.2023.112840

Tewhey, R., Kotliar, D., Park, D. S., Liu, B., Winnicki, S., Reilly, S. K., Andersen, K. G., Mikkelsen, T. S., Lander, E. S., Schaffner, S. F., & Sabeti, P. C. (2016). Direct Identification of Hundreds of Expression-Modulating Variants using a Multiplexed Reporter Assay. *Cell*, *165*(6), 1519-1529. https://doi.org/10.1016/j.cell.2016.04.027

Ulirsch, J. C., Nandakumar, S. K., Wang, L., Giani, F. C., Zhang, X., Rogov, P., Melnikov, A., McDonel, P., Do, R., Mikkelsen, T. S., & Sankaran, V. G. (2016). Systematic Functional Dissection of Common Genetic Variation Affecting Red Blood Cell Traits. *Cell*, *165*(6), 1530-1545. https://doi.org/10.1016/j.cell.2016.04.048
